# PD34 Ethics In Health Technology Assessment – A Case Study Of Prosthetic Care And Proposal For An Empirical Approach

**DOI:** 10.1017/S0266462324002976

**Published:** 2025-01-07

**Authors:** Martina Baumann

## Abstract

**Introduction:**

Medical devices cause significant spending in healthcare systems and provide methodological challenges for health technology assessment (HTA). This poster presents the results of a dissertation on the HTA of prosthetic limb technology and care, with a focus on the ethical implications of this aspect of health care. It is also a case study for ethics in HTA more generally, which will contribute to methodological discussions in this field.

**Methods:**

The methodology was based on Hofmann´s Socratic approach and empirical ethics. Literature reviews of HTA reports and ethics and social science literature on limb prosthetics and care were supplemented by semi-structured interviews with 16 prosthesis wearers and 18 stakeholders (e.g., insurance payers and orthopedic technicians) from Germany. This material served to identify and prioritize ethical issues and dive deeper into the causes of unequal access to prosthetic limbs. Beauchamp and Childress´ principlism and Norman Daniels’ theory of just health were used to describe ethical requirements and conflicts and to discuss the limits of normative recommendations that HTA can provide for decision-makers.

**Results:**

There were 42 ethical aspects related to limb prosthetic technology and care identified, reflecting also general challenges for HTA like artificial intelligence in health and resource scarcity in times of multiple crises. The perspectives of patients and stakeholders provided evidence for unequal access to limb prosthetics that was dependent on socioeconomic status, age, and living region. This is due to a combination of legal framework conditions (not supporting evidence-based reimbursement), socioeconomic success factors in interaction with gatekeepers, non-optimal quality of care (due to lack of data use and scarcity of professionals), and political non-willingness to address rationing–as well as the lack of HTAs.

**Conclusions:**

Access issues in prosthetic care and their implications for patient wellbeing, efficiency, and sustainability may be generalizable to a certain extent to other medical device types and healthcare systems. Governments should provide resources, and synergies with health services research could be leveraged to enable HTA to address the challenges of medical device and ethical assessment.

